# Explosion of Saltpetre

**Published:** 1845-11

**Authors:** 


### EXPLOSION OF SALTPETRE.

Many of our readers probably are aware that at the recent great fire in New York, a violent explosion occurred, by which the conflagration was rapidly extended and great damage caused. As gunpowder is not permitted, under an ordinance of the city, to be stored in any amount within the limits of the corporation, and as it has been stoutly denied by the occupants of the premises where the explosion occurred that there was any on the ground, much speculation and inquiry has been invoked to account for the circumstance. It is admitted, we believe, that there was a large quantity of Saltpetre there, and the opinion having been advanced that when heated to a high degree, it will explode, especially when in contact with water, the subject has been submitted to the judgment of a number of chemists, and the following, which we copy from a morning paper, appears to be the result—if result it may be called where nothing is decided:

" *Explosion of Saltpetre.*—The Committee appointed by the Common Council of New York, conclude their report relative to the explosion of saltpetre as follows:

That as far as conviction can be afforded by human testimony, your committee have had entire demonstration that there was not in the building of Crocker & Warren at the time of the explosion, or at any time anterior, any gunpowder or other substance usually deemed explosive, and that if the explosions did not result from saltpetre or its combinations with other materials, no cause for the explosions can be discovered.

Of the scientific gentlemen who have investigated the subject, the following are of opinion that Saltpetre will explode, viz:—Messrs. Hays, of Roxbury; Silliman, New Haven; Dana, Boston; Durant, Jersey City; and Hare of Philadelphia.

On the contrary:—Messrs. Renwick, Chilton, Curry, Draper and Ellett, of this city, who made the experiments."

